# Estimation of the Loading Dose for Target-Controlled Infusion of Dexmedetomidine. Reply to Eleveld et al. Comment on “Morse et al. A Universal Pharmacokinetic Model for Dexmedetomidine in Children and Adults. *J. Clin. Med.* 2020, *9*, 3480”

**DOI:** 10.3390/jcm10143004

**Published:** 2021-07-06

**Authors:** James D. Morse, L. Ignacio Cortinez, Brian J. Anderson

**Affiliations:** 1 Department of Pharmacology & Clinical Pharmacology, University of Auckland, Park Road, Auckland 1023, New Zealand; j.morse@auckland.ac.nz; 2 Department of Anesthesiology, School of Medicine, Pontificia Universidad Católica de Chile, Santiago de Chile 8331150, Chile; icortinez@gmail.com; 3 Department of Anaesthesiology, University of Auckland, Park Road, Auckland 1023, New Zealand

The parameters for a three-compartment model described by Morse and colleagues [1] were estimated from a pooled population of neonates, children, and adults. Covariate analysis included age, size, and the impact of fat mass. Hannivoort and colleagues [2] investigated the pharmacokinetics (PK) in 18 healthy adults (20 to 70 years, 51 to 110 kg, and Body Mass Index (BMI) 20.6 to 29.3 kg/m^2^). Dexmedetomidine was administered using a well-considered informative study design that involved target-controlled infusion with increasing target concentrations: 1, 2, 3, 4, 6, and 8 ng/mL—arterial plasma concentrations that often exceed those used clinically. Both the Morse [1] and Hannivoort [2] parameter sets nicely describe the observations from their cohort populations. The Hannivoort parameters used allometric theory for size (with total body weight), which adequately described published observations in children older than 2 years (i.e., after maturation of clearance).

One difference between the two parameter sets is the estimate for the volume in the central compartment (V1). Hannivoort and colleagues estimated a V1 of 1.78 L/70 kg in their cohort. Morse and colleagues estimated a much larger V1 of 25.2 L/70 kg. Although the larger V1 is consistent with most previous studies in adult patients, there is concern about the use of this V1 parameter to determine a loading dose to rapidly achieve a target concentration during target-controlled infusion. 

Classical teaching is that loading dose can be calculated for a one-compartment model by simply using volume of distribution (V) and target concentration (C_TARGET_).

$$\mathrm{Loading}\;\mathrm{Dose}=V\;\times {\;C}_{\mathrm{TARGET}}$$

However, this calculation is not applicable to many of the anesthetic drugs that are characterized using multi-compartment models. It is not solely V1 that determines dose. The other pharmacokinetic parameters used to describe disposition also have an impact on dose determination. In addition, loading dose is tempered by concentration-related adverse effects. Dexmedetomidine, unlike other anesthesia drugs used in target-controlled infusions, is usually administered slowly in children over 10–30 min to ameliorate higher concentrations associated with bradycardia or blood pressure fluctuation. The dexmedetomidine loading doses suggested by Morse and colleagues [1] are given over thirty minutes because the rate of infusion in children is not determined by an empiric adult constraint (e.g., 6 mcg/kg/h), but rather by the target concentration and concentration–adverse effect profile.

The use of a small central volume (V1) in a multicompartment PK model with a fixed loading dose may cause high concentrations above target, while the use of the larger volume of distribution at steady state (Vss) for the same loading dose may result in concentrations below target. The loading dose is used to target a concentration at the effect site, not plasma, and there is a time delay between peak plasma concentration and peak concentration at the effect site. The time to peak effect (T_PEAK_) is dependent on clearance and effect site equilibration half-time (T_1/2_keo). At a submaximal dose, T_PEAK_ is independent of dose. At supramaximal doses, maximal effect will occur earlier than T_PEAK_ and persist for a longer duration because of the shape of the pharmacodynamic (PD) concentration–response model. The T_PEAK_ concept has been used to calculate optimal initial bolus dose [3]. The volume of distribution (Vpe) at the time of peak effect site concentration (C_PEAK_) is calculated and used as follows:

$$\mathrm{Vpe}=\frac{\mathrm{Dose}}{\mathrm{Concentration}\left(T_{\mathrm{PEAK}}\right)}$$

Loading dose can then be calculated as follows:

$$\mathrm{Loading}\;\mathrm{Dose}=C_{\mathrm{PEAK}}\times \;\mathrm{Vpe}$$

These concepts can be illustrated using PKPD simulation (Figure 1). Parameter estimates for the PK model were from Morse and colleagues [1]. The PD model for blood pressure was from Potts and colleagues [4]. The PD model for heart rate was from Perez-Guille and colleagues [5]; taken from children given dexmedetomidine while undergoing ambulatory surgery. An adult concentration–sedation model described by Li and colleagues [6] was used as an illustration of sedation scoring. Rapid administration of dexmedetomidine 0.4 mg/kg in a 5-year-old (20 kg) child can achieve acceptable sedation, but it is at a cost of hemodynamic changes associated with pulse falls and blood pressure rises. The volume of distribution at the time of peak effect site concentration (Vpe) is 0.68 L/kg for this dose given rapidly over 1 min. When dexmedetomidine is administered over 30 min, the sedation score is lower and adverse effects are fewer. A Vpe of 1 L/kg can be used to determine a satisfactory infusion rate over 30 min. The infusion over 10–30 min reduces the impact V1 has on early concentrations because there is time during administration for redistribution of the drug; all parameter estimates (CL, V1, Q2, V2, Q3, V3) have influence on the observed plasma concentration. Consequently, PK parameter sets with a smaller V1 are not necessarily more suitable for target-controlled infusion (TCI). The most appropriate models for TCI are those with good predictive capabilities within the target population. It is our opinion that default settings of TCI pumps should consider the PD properties of the drug, the infusion duration, and the PK model used to drive administration.

There is variability associated with all PK parameters. Some of that variability can be reduced by incorporating covariate information (e.g., size, age, fat mass) into target-controlled infusion pumps. However, such covariate knowledge may only contribute 50% of the observed concentration variability [7]. There is also variability associated with PD parameters. Target-controlled infusion pumps provide a dosing guide. Clinical experience and unmeasurable anesthesia practitioner skill will continue to provide essential information about dosing at the patient interface.

## Figures and Tables

**Figure 1:**
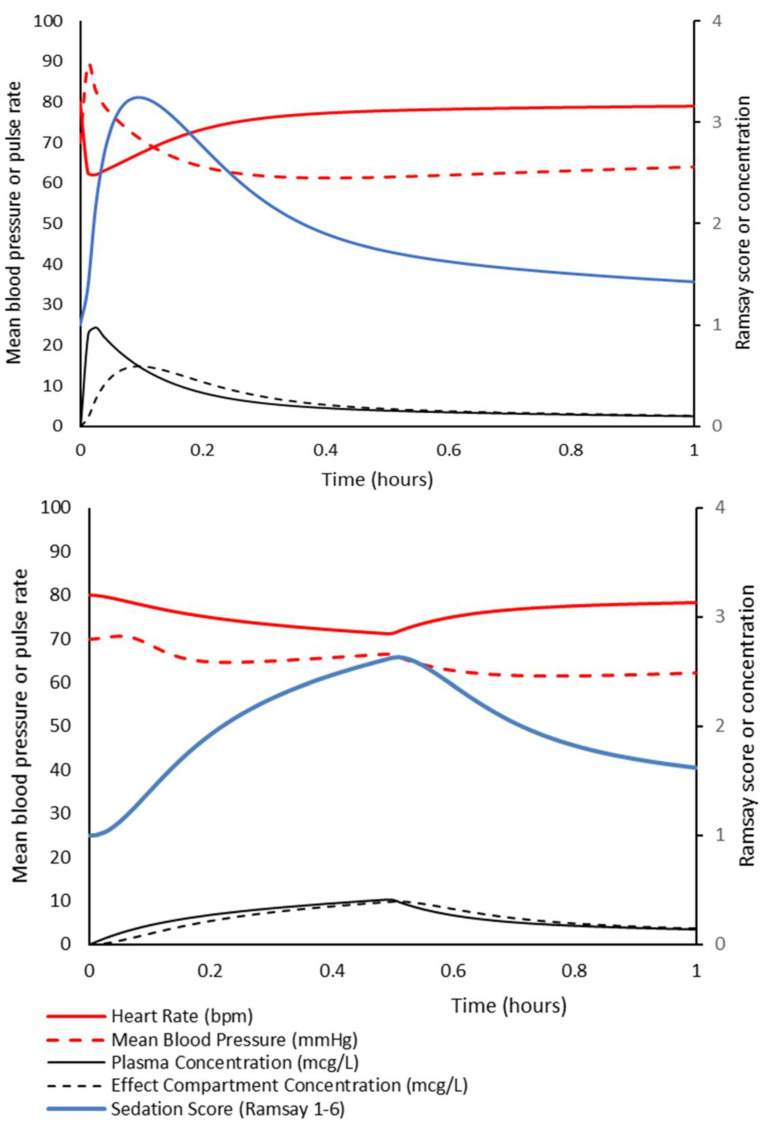
Illustrative simulation of dexmedetomidine 0.4 mg/kg given to a 5-year-old child. The drug in the upper panel was given as a rapid bolus. The lower panel shows the same dose administered as an infusion over 30 min.
